# Cross-continental emergence of *Nannizziopsis barbatae* disease may threaten wild Australian lizards

**DOI:** 10.1038/s41598-020-77865-7

**Published:** 2020-12-01

**Authors:** Nicola R. Peterson, Karrie Rose, Stephanie Shaw, Tim H. Hyndman, Lynne Sigler, D. İpek Kurtböke, Josh Llinas, Bethan L. Littleford-Colquhoun, Romane Cristescu, Celine Frère

**Affiliations:** 1 School of Science, Technology and Engineering, University of the Sunshine Coast, Sippy Downs, QLD 4556 Australia; 2 Australian Registry of Wildlife Health, Taronga Conservation Society Australia, Mosman, NSW 2088 Australia; 3 University of Queensland, Avian and Exotic Pet Service, Gatton, QLD 4343 Australia; 4 School of Veterinary Medicine, Murdoch University, Murdoch, WA 6150 Australia; 5 Faculty of Agricultural, Life, and Environmental Sciences, University of Alberta, Edmonton, AB T6G 2P5 Canada; 6 The Unusual Pet Vets, Jindalee, QLD 4074 Australia

**Keywords:** Fungi, Infectious diseases, Infectious-disease diagnostics, Ecology, Biodiversity

## Abstract

Members of the genus *Nannizziopsis* are emerging fungal pathogens of reptiles that have been documented as the cause of fatal mycoses in a wide range of reptiles in captivity. Cases of severe, proliferative dermatitis, debility and death have been detected in multiple free-living lizard species from locations across Australia, including a substantial outbreak among Eastern water dragons (*Intellagama lesueurii*) in Brisbane, Queensland. We investigated this disease in a subset of severely affected lizards and identified a clinically consistent syndrome characterized by hyperkeratosis, epidermal hyperplasia, dermal inflammation, necrosis, ulceration, and emaciation. Using a novel fungal isolation method, histopathology, and molecular techniques, we identified the etiologic agent as *Nannizziopsis barbatae*, a species reported only once previously from captive lizards in Australia. Here we report severe dermatomycosis caused by *N. barbatae* in five species of Australian lizard, representing the first cases of *Nannizziopsis* infection among free-living reptiles, globally. Further, we evaluate key pathogen and host characteristics that indicate *N. barbatae*-associated dermatomycosis may pose a concerning threat to Australian lizards.

## Introduction

Emerging fungal diseases have been associated with catastrophic declines and extinctions among wildlife and pose an increasingly recognized threat to ecosystems and biodiversity^1–3^. Ectothermic taxa are particularly vulnerable to fungal pathogens^4^ and in recent decades, herpetofauna have been severely impacted by emergent fungal diseases^5,6^. Since the late 1990s, there have been increasing reports globally of severe mycoses in reptiles caused by fungal pathogens belonging to the genera *Nannizziopsis, Paranannizziopsis*, and *Ophidiomyces* (order Onygenales); formerly members of the *Chrysosporium* anamorph of *Nannizziopsis vriesii* (CANV) complex^7,8^. Members of the genus *Nannizziopsis* have been documented as the cause of cutaneous and systemic mycoses in a diverse range of captive reptiles^9–20^, and occasionally, humans, however the species involved are distinct^8,21^. Reptile-associated *Nannizziopsis* species are contagious, primary pathogens capable of causing disease for which there is little effective treatment and usually progresses to fatality^7,22,23^. All previously documented cases of *Nannizziopsis*-associated mycoses in reptiles have been in captive animals^7–11,13,14,18–20^.

In 2013, two free-living Eastern water dragons (*Intellagama lesueurii*; EWDs) from locations separated by 30 km across Brisbane (Queensland, Australia) were identified with extensive yellow to tan/brown proliferative dermal lesions, lethargy, and emaciation. Between 2016 and 2017, ecologists conducting research in a population of EWDs in a Brisbane city park, well-studied since 2010, observed an increasing number of individuals with similar skin lesions. During a 2018 population survey at this location, 34.5% (88 of 255) of the EWDs examined presented with these characteristic skin lesions, ranging from mild and localized to severe and extensive, often accompanied by necrosis of digits and tails and an emaciated body condition. By March 2020, clinically similar cases of disease had been identified in three additional free-living lizard species from locations separated by more than 3500 km across Queensland, New South Wales, and Western Australia. Similar cases were also detected in a group of captive lizards in Victoria. Faced with a widespread, unknown disease threatening Australian lizards, we conducted an investigation to describe the syndrome and identify the etiologic agent. Herein, we characterize an emerging dermatomycosis caused by *Nannizziopsis barbatae* (formerly *N. barbata*^24^) in five Australian lizard species, representing the first confirmed cases of *Nannizziopsis* infection among free-living reptiles globally. Further, we describe a novel isolation method for enhanced recover of the fungus and evaluate key pathogen and host characteristics that indicate *N. barbatae*-associated dermatomycosis may pose a serious threat to Australian lizards.

## Results

Twelve severely affected free-living lizards, representing four species and eight locations across Australia were examined; this included nine EWDs and a tommy roundhead dragon (*Diporiphora australis*) from six widely-separated sites across the Brisbane region, an Eastern blue tongue skink (*Tiliqua scincoides scincoides*) from Dubbo, New South Wales, and a shingleback skink (*Tiliqua rugosa*) from Perth, Western Australia (Supplementary Fig. S1). A Centralian blue tongue skink (*Tiliqua multifasciata*), one of five affected animals housed in a private collection in Victoria, was included in the study to determine whether a common pathogenic agent was associated with skin lesions in both wild and captive lizards. In all 13 cases, infection with the fungal pathogen *Nannizziopsis barbatae* was confirmed by histopathology, culture, and/or PCR and DNA sequencing (Supplementary Tables S1, S2).

**Figure 1:**
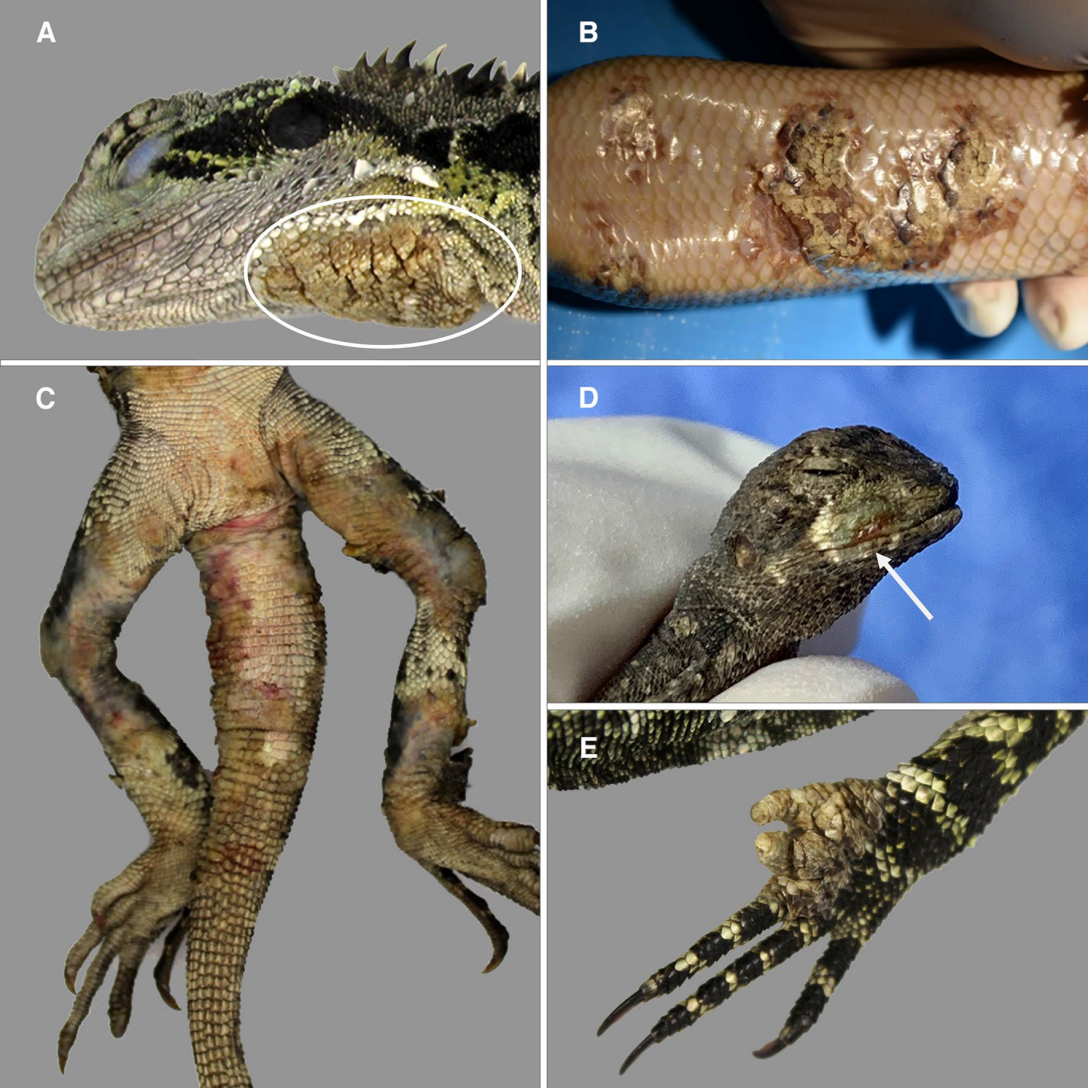
Gross pathology of Australian lizards with severe dermatomycosis caused by *Nannizziopsis barbatae* (**A**) Free-living Eastern water dragon (white circle indicates skin lesion; case EWD006) and (**B**) captive Centralian blue tongue skink (case CBT001) with proliferative skin plaques [Photo credit: Dr. S. Simpson]. (**C**) Free-living Eastern water dragon (case EWD005) and (**D**) tommy roundhead dragon (case TRH001) with ulcerative skin lesions (**E**) free-living Eastern water dragon (case EWD006) with digital necrosis and loss.

Gross presentation of affected animals included severe, proliferative yellow to tan/brown crusted lesions which ranged in coverage of 10–60% of the overall skin surface (Fig. 1A–C, Supplementary Table S3). In some cases, surface crusts readily sloughed from lesions, exposing ulcerated, erythematous, and bleeding skin. Ulceration, necrosis, and loss of digits were common (Fig. 1D,E). Affected lizards also presented with lethargy and moderate to profound emaciation (Supplementary Fig. S2, Table S3). Treatment was initiated for three of the affected lizards (Supplementary Table S3). One failed to improve and was euthanized (EWD001) and another died during treatment (CBT001). While the Eastern blue tongue lizard (EBT001) had a resolution of skin lesions following surgical debridement, removal of an affected limb, and topical antimycotic therapy, the animal was released to the wild prior to confirmation of *Nannizziopsis* infection and subsequently lost to follow up.

**Figure 2:**
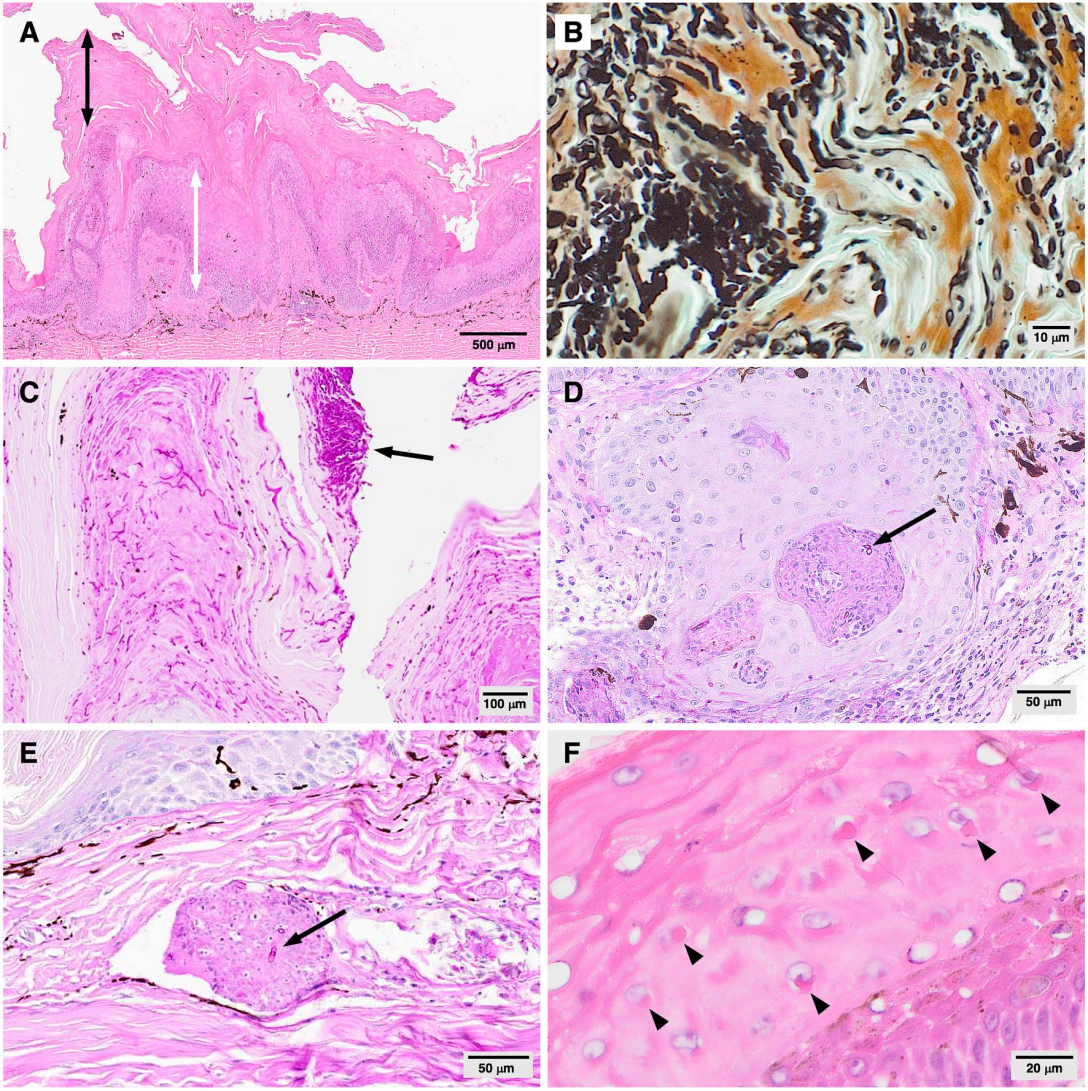
Histopathological features of cutaneous lesions from free-living Australian lizards with *Nannizziopsis barbatae*-associated dermatomycosis (**A**) severe hyperkeratosis (black double pointed arrow), papillary epidermal hyperplasia (white double pointed arrow) (case EWD009; H&E). (**B**) Abundant fungal hyphae and arthroconidia, morphologically consistent with *Nannizziopsis* spp. (Case EWD009; GMS) (**C**) surface tuft of arthroconidia (arrow) (case EWD009; PAS). (**D**) Dysplastic invaginating epithelium with intralesional fungal hyphae (arrow) (case TRH001; PAS). (**E**) Perivascular dermal granuloma with intralesional hyphae (arrow) in the sole animal with deep dermal mycosis (case EWD008; PAS). (**F**) Epidermal intracytoplasmic inclusion bodies (arrowheads) (Case EWD009; H&E).

Consistent histopathological findings of affected skin included moderate to severe hyperkeratosis, papillary epidermal hyperplasia, and moderate to severe epidermal necrosis and ulceration (Fig. 2A, Supplementary Table S4). Presentation differed somewhat between species; skin lesions in the EWDs and tommy roundhead dragon were more proliferative in comparison to those of the Centralian blue tongue lizard and shingleback skink, which were more severely ulcerative and necrotizing. Although bacteria were observed on the skin surface in some cutaneous lesion sections, morphologically consistent fungal elements were present in all skin lesion sections examined from all lizards. Abundant, alternately branching fungal hyphae were visible within the keratin and epidermal layers (Fig. 2B); arthroconidia, the presumed propagules of infection^8,22^, occurred in large numbers, occasionally in vast aggregates on the skin surface (Fig. 2C). Fungal elements penetrated the epidermis to the level of the stratum spinosum, frequently in association with downward migration of the epithelial layer and occasionally extending to deep invaginations of the epithelium containing granulomas and heterophil aggregates (Fig. 2D). Ulceration, hemorrhage, and inflammation were noted at the dermo-epidermal junction, and although fungal elements were largely restricted to the keratin and superficial epidermal layers, the underlying dermis exhibited a range of multifocal inflammatory changes including edema, pigmentary incontinence, and variable infiltrates of heterophils, lymphocytes, plasma cells, and macrophages. In the most clinically severe case, the presence of a perivascular dermal granuloma with intralesional fungal hyphae within a section of necrotic digit (Fig. 2E) indicated a potential for systemic spread, however, there was no evidence of fungal invasion beyond the dermis in any of the cases examined.

No acid fast or partially acid fast microorganisms were detected in skin lesion sections, nor was there evidence of bacteria other than those superficially coating the surface of the keratin layer. Rare, localized, eosinophilic epidermal intracytoplasmic inclusion bodies were observed in skin sections from six EWDs (Fig. 2F). These inclusions may suggest a concurrent viral infection or may represent host protein; skin samples from these animals were PCR-negative for poxvirus and papillomavirus. Five lizards in this study had evidence of renal disease including tubular necrosis and glomerulonephropathy, possibly associated with dehydration, ischemia, and/or glomerulopathy secondary to chronic inflammation.

Fungal isolation using standard methodology, whereby cutaneous lesion samples were plated directly onto selective media^25^, yielded mixed results in this study due to the presence of rapidly growing contaminating fungi. An improved method of fungal isolation was devised by modifying a conventional serial dilution technique, often used to isolate microorganisms from soil^26^. Using this method, fungal isolates were successfully obtained from dilutions of affected cutaneous tissue suspended in sterile deionized water.

**Figure 3:**
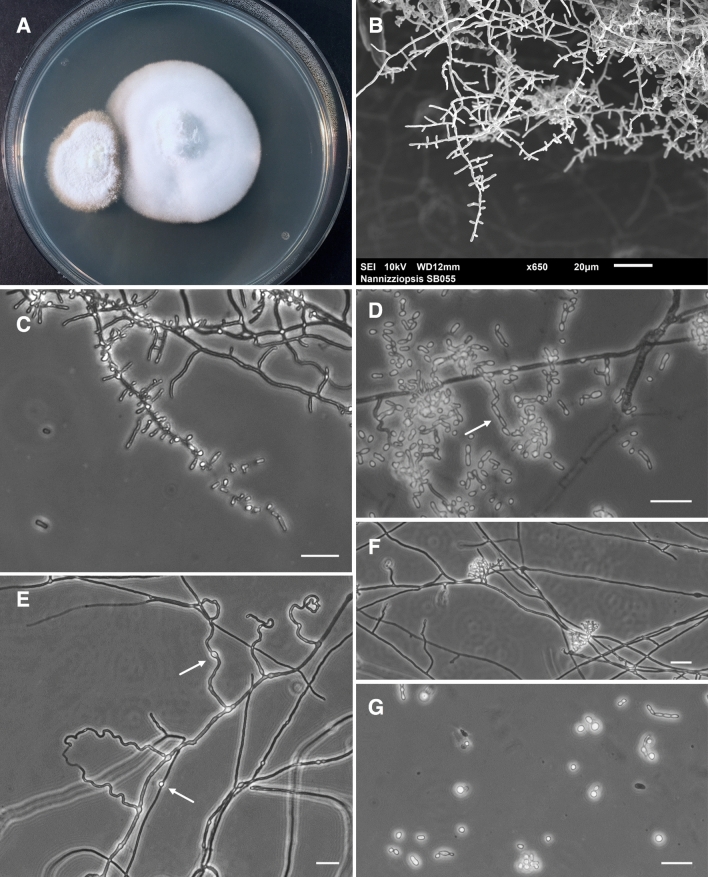
Morphological characteristics of *Nannizziopsis barbatae*, isolated from cutaneous lesions of free-living Eastern water dragons and incubated on PDA at 30 °C. (**A**) Colony morphology on PDA at 21 days. (**B**) Scanning electron micrograph showing branched fungal hyphae bearing aleurioconidia. (**C**) Microscopic morphology showing pyriform to clavate, mainly sessile aleurioconidia, 2.5–10 μm in length and 1.5–3 μm in width, born along septate branching hyphae and occasionally subtended by swollen cells. (**D**) Fission arthroconidia, 2.8–8.3 μm in length and 1.8–3.3 μm in width. (**E**) Abundant undulate hyphae, characteristic of *Nannizziopsis* species^8^, and intercalary chlamydospores (arrows). (**F**) Ascomatal initials and (**G**) budding cells were occasionally present. Bars 20 μm.

A yellowish-white fungus morphologically consistent with *Nannizziopsis* species was isolated from cutaneous lesion samples of 11 affected lizards (Supplementary Table S1). Isolates grown on PDA and incubated at 30 °C for 21 days formed powdery to cottony, slightly raised colonies that ranged from slightly to moderately zonate (Fig. 3A). Microscopic morphology of isolates (Fig. 3B–G) and an absence of growth at 35 °C (Supplementary Fig. S3) were largely consistent with that of the type strain of *N. barbatae*^8,20^. However, intercalary chlamydospores (Fig. 3E) were frequently observed and are a notable finding, as this feature has not previously been described for *N. barbatae* or any *Nannizziopsis* species except *N. chlamydospora*^8,27^. While the presence of ascomatal initials (Fig. 3F) suggests the potential for sexual reproduction, this was not observed in these isolates, nor has it been reported in any other reptile-associated *Nannizziopsis* species to date.

Other organisms cultured from skin lesions varied by sample. Fungal culture yielded primarily *Candida*, *Aspergillus*, and *Fusarium* species. Bacterial culture from cutaneous lesions of three lizards yielded variable, mixed growth including *Enterobacter cloacae*, *Staphylococcus epidermidis*, and *Strenotrophomonas maltophila* (Supplementary Table S5).

DNA sequencing of a 220–559 bp fragment of the internal transcribed spacer (ITS) region obtained from fungal isolates and skin biopsies showed 98.6–100% nucleotide similarity to the corresponding regions of *N. barbatae* (GenBank accession number: JF323871; type strain UAMH 11185), previously identified as the cause of fatal, systemic mycosis in a captive coastal bearded dragon (*Pogona barbata*) in Australia^8,20^ (Supplementary Table S2). Currently, there are no publicly available sequences in the 28S rDNA or β-tubulin gene regions from the type strain of *N. barbatae*, so comparisons to this species could not be made for these genomic regions. 28S rDNA sequence fragments obtained from isolates in this study were 524–527 bp in length and showed 99% nucleotide similarity to *N. crocodili* (GenBank accession number: MT478064), recently isolated in Australia from captive freshwater crocodiles with mycotic dermatitis^9^. DNA sequencing of a 373 bp fragment of the β-tubulin region obtained from fungal isolates was 87.7–88.5% similar to the cognate region of *N. pluriseptata* (GenBank accession number: HF547890)^27^. Although there was little sequence variability between isolates, they were not 100% identical at any of the loci examined; ITS sequence fragments differed by either 1 or 2 nucleotides between isolates; 28S fragment sequences differed by 1 nucleotide; and β-tubulin fragment sequences differed by 1, 2, or 3 nucleotides. Sequence differences did not appear to be related to host species or location.

**Figure 4:**
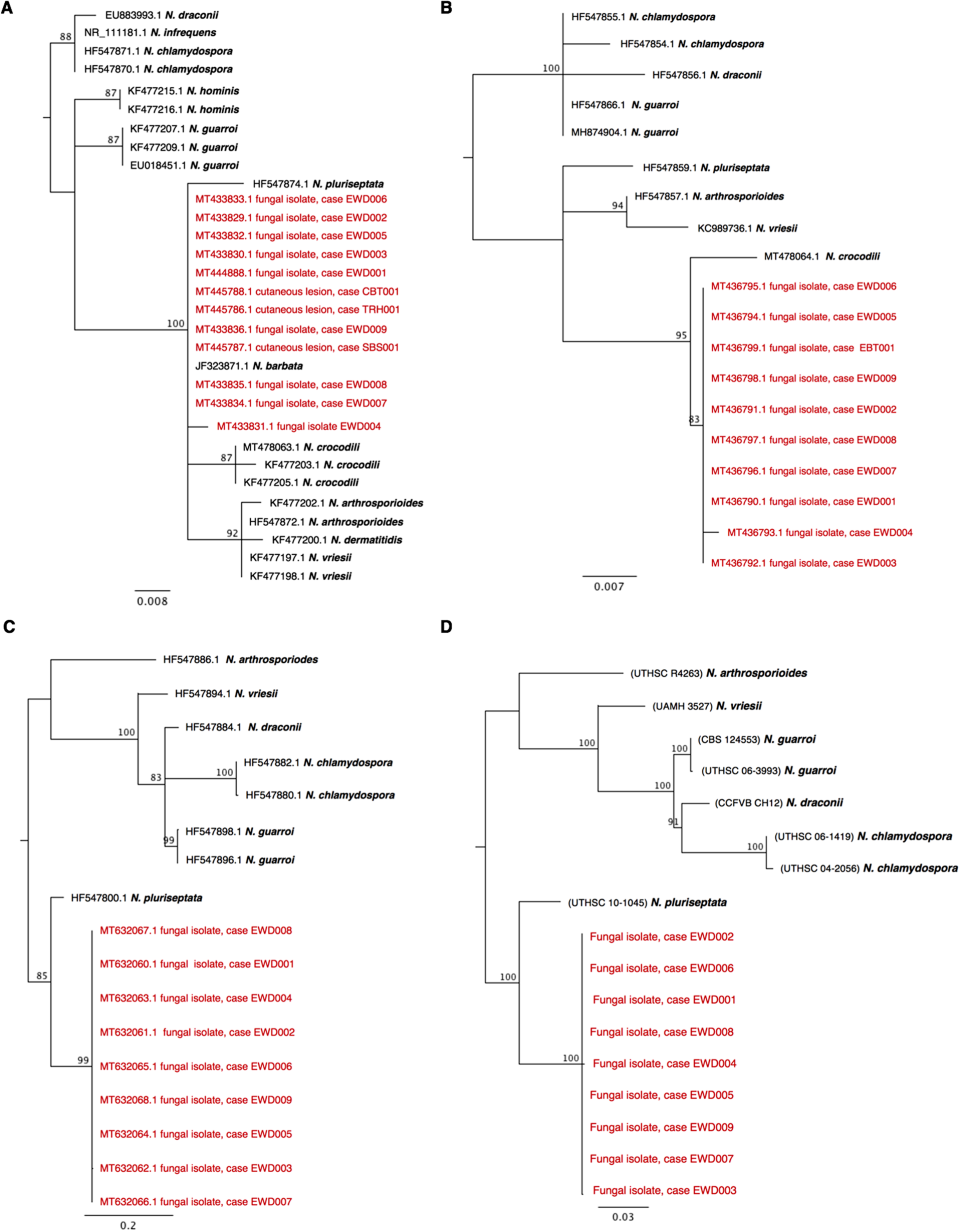
Maximum likelihood trees depicting the relationship of fungal isolates obtained in this study with other members of the *Nannizziopsis* genus for which similar sequences were available. Analyses were performed using (**A**) ITS2, (**B**) 28S, (**C**) β-tubulin, and (**D**) concatenated (ITS + 28S + β-tubulin) sequence alignments. Midpoint-rooted consensus trees were constructed from 750 original trees produced using PhyML, based on a generalized time reversible substitution model, bootstrapped with 1000 random re-samplings of the data. Branches are supported by consensus support threshold values > 75%. Scale bars represent the mean number of nucleotide substitutions per length.

Maximum likelihood analysis of a 220 bp ITS2 sequence alignment showed the isolates obtained in this study grouped with *N. barbatae* in a well-supported clade, most closely associated with *N. crocodili* and *N. pluriseptata* (Fig. 4A). Nucleotide sequences from this ITS2 fragment did not provide adequate resolution to distinguish between all *Nannizziopsis* species, however phylogenetic analyses of 28S rDNA, β-tubulin, and concatenated alignments supported similar grouping of the isolates (Fig. 4B–D).

A search of the Australian Registry of Wildlife Health database identified 1232 squamates examined grossly and histologically between 2000 and 2020, as well as 610 squamates admitted to the Taronga Wildlife Hospital for examination by wildlife veterinarians. The first cases of *N. barbatae* infection were detected in 2008 and 2009^8,20^. Since 2010, fungal culture was conducted from specimens of 140 squamates with no detection of *N. barbatae* from any animal, apart from the cases reported here.

## Discussion

*Nannizziopsis* species have been documented as the cause of severe, often fatal disease in a wide range of captive reptiles, including several reports concerning captive Australian reptiles^7–10,20^. *Nannizziopsis barbatae* has been isolated only once previously from a captive coastal bearded dragon in Australia with deep dermal and systemic mycosis^20^. Here we provide evidence that cases of severe dermatitis, debility, and death in five Australian lizard species from locations across the continent were caused by *N. barbatae*, representing the first cases of *Nannizziopsis* infection among free-living lizards, globally. *Nannizziopsis*-associated mycoses in captive lizards are often reported as dermally invasive and sometimes multi-systemic^8,19,20,28^. Among the lizards investigated here, the presence of *N. barbatae* within skin lesions was associated with a spectrum of histopathologic changes and included hyperkeratosis, epidermal hyperplasia, ulceration, and necrosis. These changes differed slightly between the species examined here, raising the possibility that presentation could vary across host species and environments. However, this was based on single cases and further investigations may come to different conclusions. The most severely affected EWD presented with deep dermal mycosis, however in every other lizard examined histologically, fungal elements were limited to the keratin and epidermal layers. Skin lesions were grossly similar to those previously described for captive lizards with *Nannizziopsis*-associated dermatomycoses^20^, however tended to be more extensive in these free-ranging animals. Most of the affected lizards were emaciated and several showed signs of dehydration, renal disease, and gout; a presentation similar to that reported for freshwater crocodiles with dermatomycosis caused by *N. crocodili*, where infection was limited to the superficial layers of the skin and visceral gout was identified as the main pathology contributing to death^9^. Similarly, among snakes with snake fungal disease, caused by the related onygenalean pathogen, *Ophidiomyces ophidiicola* (formerly ‘ophiodiicola’^29^), infection may be limited to the epidermis and associated with anorexia and an emaciated body condition; death may result from secondary sequelae, rather than the infection *per se*^6,30^.

*Nannizziopsis* species have been shown to act as primary pathogens^22^ and are capable of causing severe disease in otherwise healthy captive reptiles kept under optimal conditions^18,22^. It has been suggested that poor husbandry may contribute to the development of disease among reptiles in captive settings^10,20^. Similarly, it is possible that a range of factors such as suboptimal environmental conditions, poor pre-existing health, or the presence of other infectious agents, might play a role in the emergence and presentation of this disease in free-living lizards. Most of the affected lizards examined in this study were inhabitants of moderately to highly urbanized environments. Indeed, urbanization has been associated with negative impacts on wildlife health, including higher parasite loads, increased physiological stress, and greater likelihood of disease transmission^31^. It is possible that the health of some lizards evaluated in this outbreak was compromised by pre-existing factors, which may have increased their vulnerability to *N. barbatae* or contributed to the severity of disease. Equally, *N. barbatae* infection might have rendered hosts more susceptible to secondary disease processes. We observed intracytoplasmic inclusions within the dermis of six EWDs investigated here. While these inclusions could represent host protein or keratohyaline granules, they may indicate a concurrent viral infection. We undertook specific tests to rule out the presence of viruses associated with eosinophilic inclusions in reptiles, however we could not exclude the possibility of co-infection with other pathogens. Additional work is required to examine the complex interplay between environment, host, and microbe, as well as the potential role of co-pathogens. Nonetheless, although the presence of other microorganisms was noted in some animals, *N. barbatae* was the only organism consistently identified in skin lesions from all affected lizards in this study.

The natural ecology of *Nannizziopsis* species is largely unknown. Among reptiles, *Nannizziopsis* species have so far been associated exclusively with diseased tissues^7^. A culture-based survey of skin samples obtained from actively shedding, healthy captive reptiles revealed a wide range of fungal species but did not detect any members of the genus *Nannizziopsis*, suggesting that these fungi are not part of the normal skin mycobiome^32^. Although a carrier state has been suggested^9,19^, this has not yet been studied explicitly. Molecular screening of both healthy and affected lizards in free-living populations affected by *N. barbatae* will help to elucidate the relationship between clinical signs and the presence of *Nannizziopsis* species.

*Nannizziopsis barbatae* may have been introduced to wild lizards through spillover or may be an endemic fungus only recently detected or emerging among wild lizards due to altered host susceptibility or changing environmental conditions. There have been no reports of *N. barbatae* outside of Australia and the isolates obtained here were not 100% identical to each other within the genetic loci examined, which may suggest endemism. However, genetic variability between isolates was low and did not appear to be related to host species or geographic location. For example, the ITS sequence fragment obtained from one of the EWD isolates from Brisbane (EWD004) was more similar to that from the isolate obtained from a shingleback skink located 3500 km away in Perth (SBS001) than it was to all other Brisbane EWD isolates. We were unable to obtain sequences at all three loci for all isolates, so our ability to compare *N. barbatae* isolates across host species and geographic locations was limited. Further molecular characterization is required to explore the possible origins of *N. barbatae*.

The rapid spread of disease among free-living EWDs in Brisbane suggests that *N. barbatae* has the potential to negatively impact free-living lizard populations. Moreover, the presence of *N. barbatae*-associated dermatomycosis in free-ranging lizards exhibits key characteristics that are associated with emerging infectious diseases that threaten biodiversity: high pathogenicity and contagiousness, likely persistence in environmental reservoirs, a prolonged period of disease, little effective treatment, and a broad host range^2,33,34^.

Reptile-associated *Nannizziopsis* species are highly pathogenic organisms, causing significant morbidity and mortality among infected hosts^7,22^. In captive settings, disease can spread rapidly between animals in close proximity and has been associated with severe epizootics^10,13^. Transmission is believed to occur directly through contact with infected individuals, and indirectly via fomites^22^. In an experimental infection trial, Paré et al.^22^ showed that *N. dermatitidis* could be isolated from contaminated cages and shed skin of infected lizards, and that host exposure to erumpent arthroconidia from skin lesions leads to infection.

The potential for persistence and transmission of reptile-associated *Nannizziopsis* species within the natural environment requires investigation. It is likely that *N. barbatae* arthroconidia persist on the shed skin of infected lizards, and we suspect that this material acts as a source of infection. Whether *N. barbatae* can persist within other environmental substrates, such as soil, is unknown. Physiological study of isolates, environmental sampling, and screening for the presence of asymptomatic carriers is required, as these may represent important reservoirs of infection.

Captive reptiles infected with *Nannizziopsis* species often experience a prolonged duration of disease, with a progression of clinical signs over a period of weeks or months^19,28,35^. The infection can be readily transmitted to individuals in close proximity during that time^10,13^. One free-living lizard investigated here (case EWD005) was first identified with suspect skin lesions two years prior to the current investigation and showed a progressive decline in body condition over that period. Fungal hyphae and vast numbers of arthroconidia were present at the surface of skin in histologic sections from all lizards examined in this study. This viable infectious material sloughs from the skins of diseased lizards^22^, and a lengthy period of disease increases the opportunity for transmission to other individuals.

Attempts to treat *Nannizziopsis*-associated dermatomycoses in reptiles with antimycotic therapy and surgical measures have had little success and infection usually progresses to a fatal outcome^23,36^. In a small number of cases in captive reptiles where treatment has resulted in the resolution of skin lesions, topical and systemic antimycotics were applied daily over a prolonged period of weeks or months^17,28^. Even after apparent resolution, skin lesions can recur following cessation of treatment^19,20^. Using currently available treatment strategies, disease management in wild reptile populations would be costly and impractical.

*Nannizziopsis* species have been documented as the cause of disease in multiple reptilian host species^9–11,13,16,25,37^. The detection of *N. barbatae* in five phylogenetically and ecologically distinct lizard species that are common and collectively distributed across much of the Australian continent indicates that this pathogen has a broad host range among lizards and is capable of establishing in hosts across a variety of climatic and environmental conditions. The five species of affected lizards in this study represent two distinct squamate families (Agamidae and Scincidae), and occur in habitats ranging from aquatic and riparian, to woodland, forest, arid grassland, and desert, as well as urban and semi-urban environments^38^. Of particular concern is the presence of this disease in the EWD, a common, widely-distributed^38^, gregarious^39^, semi-aquatic species that occurs at high population densities and is mobile through waterways^40^. Furthermore, there is potential for this pathogen to be distributed through the legal and illegal movement of reptiles, which constitute the largest portion of Australian wildlife on the global black market^41,42^.

Although *Nannizziopsis* species have been documented as the cause of disseminated disease in humans^21,43^, the species involved are different to those affecting reptiles^7,8^. *Nannizziopsis barbatae* isolates obtained in this and a prior study showed no growth at 35 °C^8^, and it is unlikely that this fungus poses a risk to human health. Nonetheless, appropriate biosafety measures should be taken by those who handle lizards with skin lesions from an unknown cause.

The isolation of *Nannizziopsis* species from clinical veterinary samples has proven challenging, as specimens are often contaminated with bacteria and soil-associated fungi. The isolation method devised for this study enabled straightforward and successful isolation of *N. barbatae* from every skin specimen for which the method was applied, including those that were known to be heavily contaminated and/or had been stored frozen for more than 12 months. This improved method provides a valuable tool for future diagnoses of *Nannizziopsis* infections in reptiles and may be applicable to a range of fungal pathogens of veterinary interest.

The widespread presence of *N. barbatae*-associated dermatomycosis in free-living Australian lizards raises serious conservation concern. Globally, it is estimated that 20% of all reptile species are now threatened with extinction^44^ due to a number of threatening processes, including disease^6,45^. As a largely understudied and often cryptic group, there is potential for population and species-level impacts to occur undetected. Reptiles are an iconic component of the Australian landscape and represent a significant portion of the vertebrate taxa in nearly all Australian terrestrial ecosystems^46^. The threat of losing a high portion of diversity and vertebrate biomass could have untold consequences for Australian ecosystems.

## Methods

The Queensland component of this research was approved by the University of the Sunshine Coast Animal Ethics Committee (ANS1858) and conducted under a Scientific Purposes Permit from the Queensland Department of Environment and Science (WISP17696616). Additional samples were collected during routine veterinary examinations under the auspices of Taronga Conservation Society Australia’s Opportunistic Sample Collection Program. All methods were carried out in accordance with the 2013 Australian National Health and Medical Research Council ‘Australian Code for the Care and Use of Animals for Scientific Purposes’.

### Collection of animals

Lizards EWD002–EWD007, and EWD009 were collected during targeted disease surveillance across Brisbane city parks. All remaining lizards were identified during routine veterinary examinations and collected opportunistically.

### Pathology

Two affected lizards died naturally and ten were anesthetized using alfaxalone (Jurox, Australia) at a dose of 10 mg/kg by intravenous (IV) injection into the caudal vein, then euthanized with pentobarbitone (Virbac, Australia) 325 mg/mL, at a dose of 150 mg/kg IV, diluted 50:50 with sterile water. An extensive post-mortem examination was conducted for each of these lizards, and all lesions were described and photographed. Skin swabs were collected for PCR testing using sterile nylon flocked swabs (Copan FloqSwabs, Copan Diagnostics, USA) premoistened with 0.9% sterile saline and rubbed in a rotating motion with moderate pressure for 10 s over cutaneous lesions. Swabs were stored frozen at − 20 °C until DNA extraction. Cutaneous lesion tissue was then collected and stored at − 20 °C for subsequent fungal isolation and molecular analysis. Samples of affected and unaffected skin and all major organs were placed in 10% neutral buffered formalin for a minimum of 7 days prior to being processed and sectioned routinely for histological examination. Paraffin embedded sections were stained with hematoxylin and eosin (H&E), mounted with a coverslip and examined by light microscopy. Pathologic findings were graded on a scale of 1 (mild) to 3 (severe). Additional representative sections were stained with Periodic acid-Schiff (PAS) and Gomori methenamine silver (GMS) stains to highlight fungal elements. Fite Faraco stain was used to exclude *Mycobacterium* species.

### Bacterial culture

Bacterial culture was performed using cutaneous lesion samples of three affected lizards. Samples were inoculated onto horse blood agar and MacConkey agar (Thermo Fisher, Scorseby, Australia) and incubated at 35 °C in 5% CO_2_. Samples were also inoculated onto anaerobic blood agar (Thermo Fisher, Scorseby, Australia) and incubated at 35 °C under anaerobic conditions. Plates were examined daily for growth for up to 10 days.

### Fungal isolation and culture

Fungal isolation from four cases (EWD001, EBT001, CBT001, SBS001; Supplementary Table S1) was conducted by placing ~ 3 × 3 mm cutaneous lesion biopsy segments directly onto Mycosel Agar containing cycloheximide and chloramphenicol (Becton Dickinson, USA), and incubating at 30 °C for 21 days. Suspect colonies were sub-cultured onto potato dextrose agar (PDA; Oxoid, USA). The same technique was attempted for additional cases but was largely unsuccessful due to overgrowth with contaminating fungi. To reduce the impact of contaminants, a new method for isolation of *Nannizziopsis* species was developed by modifying a conventional serial dilution technique^26^. Fungal isolation was performed for each case by preparing a suspension of affected cutaneous tissue in sterile water at a ratio of 1:100 by weight, and shaken at 600 oscillations per minute (Stuart SF1 Flask Shaker; Stuart Equipment, UK) for 30 min. A tenfold dilution series was then prepared from each of these stock solutions, with concentrations descending from 10^–2^ to 10^–7^. From each concentration, 200 µL was plated onto Mycosel Agar with added streptomycin sulfate at 70 ppm. Plates were incubated at 30 °C and observed daily for growth for a minimum of 14 days. Suspect colonies were sub-cultured onto PDA and incubated at 30 °C for 7–14 days, at which point morphological characteristics were examined by microscopy.

#### Fungal colony growth and morphology

Fungal isolates obtained from EWDs were grown on PDA at 30 °C for 21 days for evaluation of colony morphologies. To assess growth response at different temperatures, a subset of isolates (n = 4) was grown on PDA in triplicate at three temperatures: 25 °C, 30 °C, and 35 °C. The surface and reverse of each colony was photographed at days 7 and 28. Colony dimensions were determined from the photographs using the software package, ImageJ (version 1.52 k). Growth at each temperature was reported as the mean accumulative growth between days 7 and 28.

#### Microscopy

Morphological features of fungal isolates were examined and imaged using optical and scanning electron microscopy (SEM). Slide cultures were prepared for optical microscopy of each isolate by placing sterile glass coverslips on the surface of prepared water agar. A 5 × 5 mm plug of PDA was placed on top of each coverslip, inoculated with a single isolate, and a second glass coverslip was then placed on top. Plates were incubated at 30 °C. Glass coverslips were removed at varying time points between 9 and 38 days. Wet slide mounts were prepared using lactophenol cotton blue solution (Sigma-Aldrich, USA) and viewed using a Nikon Eclipse Ti microscope (Nikon Instruments, USA) in phase contrast mode with a 20 × objective lens. Images were collected using NIS Elements AR software (v 4.20.03).

Fungal isolates were prepared for SEM by placing ~ 5 × 5 mm sections of sterile Whatman Grade 1 cellulose filter paper (Sigma-Aldrich, USA) onto PDA plates. Each plate was inoculated with a single isolate and incubated at 30 °C for 12 days. Filter paper sections with visible fungal growth were lifted off each plate and vapor-fixed for 24 h with 4% osmium tetroxide^47^. Fixed samples were then mounted on specimen stubs and sputter coated with Au/Pd using a Q150T Plus turbomolecular pumped coater (Quorum Technologies, UK). Samples were examined and imaged using a JSM-6010LA Analytical Scanning Electron Microscope (JEOL, Japan).

### Molecular analyses

#### DNA extraction, PCR, and sequencing

DNA extraction and PCR targeting a fragment of the ITS region was conducted using diagnostic skin swabs, cutaneous lesion samples, and fungal isolate samples. Genomic DNA was extracted from skin swab samples using the Purelink Viral RNA/DNA Mini Kit (Thermo Fisher, USA) according to the manufacturer’s instructions. Swabs were vortexed (~ 30 s) in sterile saline (~ 1 mL) before a 200 µL aliquot was added to carrier RNA, a lysis buffer and proteinase K and then processed as per the manufacturer’s instructions. The QIAamp Cador Pathogen Mini Kit (Qiagen, USA) was used to extract DNA from fungal isolates. Five microliters of fungal suspension was added to 195 µL of PCR-grade water and the manufacturer’s instructions were then followed using the phenol–chloroform pre-treatment method (T4). DNA from fresh frozen skin was extracted as per the method for fungal isolates except that thawed tissue segments of ~ 3 × 3 × 3 mm were first homogenized in 400 µL of PCR-grade water and 200 µL of phenol (pH 8, Sigma, St. Louis, Missouri, USA) using a Mini-Beadbeater 24 (Biospec, USA) at 3000 oscillations per minute for 2 min using 0.5 mL of silicone-carbide sharp particles (1 mm diameter; Biospec, USA). PCR testing was conducted targeting a 261 nucleotide DNA fragment of the ITS gene of *Nannizziopsis* species, which was amplified using 10 µL of 2 × master mix from the PlatinumTM Green Hot Start PCR Master Mix (2X) (ThermoFisher, USA), forward 5′GCATCGATGAAGAACGCAGCGA and reverse 5′GGYCAGCKCCGGCCGGGTC primers at 500 nM final concentration, and 1 µL of extracted DNA, with the final volume brought to 20 µL using PCR-grade water. Thermal cycling conditions were as follows: 94 °C for 2 min, followed by 10 cycles of 94 °C for 20 s, 72 °C for 45 s (decreased by 1 °C per cycle), followed by 30 cycles of 94 °C for 20 s, 62 °C for 45 s, 72 °C for 30 s. A no template extraction was included as a negative control. Following gel electrophoresis, amplicons near in size to 261 bp were cut from the gel for Sanger sequencing using standard methods.

Samples of fungal isolates were also submitted to a commercial laboratory for further molecular characterization. PCR was used to amplify the D1–D2 domains of 28S rDNA, and β-tubulin gene regions using the primer pairs NL1/NL4^48^ and Bt2a/Bt2b^49^, respectively. Amplicons were Sanger sequenced by standard methods and consensus sequences were extracted from the aligned chromatograms.

#### Phylogenetic analyses

Consensus sequences were compared against the National Centre for Biotechnology Information (NCBI) nucleotide database using the ‘blastn’ algorithm^50^. Sequence and phylogenetic analyses of ITS, 28S, and β-tubulin sequences was conducted using Geneious Prime (version 2019.2.1). Publicly available sequences from 11 members of the *Nannizziopsis* genus were obtained from the NCBI database, GenBank^51^ (Supplementary Table S6). Included in the analyses were sequences obtained from fungal isolates of nine affected EWDs and an Eastern blue tongue skink (*Tiliqua scincoides scincoides*), and from cutaneous lesion samples collected from a tommy roundhead dragon (*Diporiphora australis*), shingleback skink (*Tiliqua rugosa*) and a Centralian blue tongue skink (*T. multifasciata*; the only captive animal included in the study).

Sequences in each dataset were aligned using ClustalW (version 2.1) with default parameters^52^. After manual checking, ITS2, 28S and β-tubulin alignments were trimmed to 220, 524, and 385 bp, respectively. A concatenated alignment (ITS2 + 28S + β-tubulin) was constructed using sequences from isolates and samples for which all three regions were available; this included nine sequences obtained in the present study, and seven that were publicly available, representing six *Nannizziopsis* species (Supplementary Table S6). Sequences for each region were aligned separately, then trimmed and concatenated. Maximum likelihood analyses were performed on these alignments with PhyML (version 2.2.4) using a generalized time reversible model, bootstrapped with 1000 random re-samplings of the data^53^. Midpoint-rooted consensus trees were constructed from 750 original trees with a 75% threshold, to gain an estimate of the level of support for each clade in the final tree^54^.

#### Viral screening

Skin samples from six EWDs with histologically detected epidermal intracytoplasmic inclusion bodies were screened for poxvirus and papillomavirus using previously described methods^55,56^.

#### Denominator data

The denominator data for this study was provided by the Australian Registry of Wildlife Health (Registry), which has a central role in the detection and diagnosis of endemic, emerging, and exotic diseases of Australian wildlife, and was directly involved in diagnosing the first cases of ‘CANV’ infection in captive coastal bearded dragons in 2009^20^, later attributed to *N. barbatae*^8^. We conducted a Registry database search for all squamate and squamate mycosis cases between 2000 and 2020, and for squamate fungal culture conducted between 2010 and 2020, after *N. barbatae* was first identified.

## Supplementary information

Supplementary Information.

## Data Availability

Sequence files have been deposited in the National Centre for Biotechnology Information database, GenBank, under accession numbers MT444888, MT445786–MT445788, MT433829–MT433836, MT436790–MT436799, and MT436790–MT436799. Fungal isolates have been deposited with the Centre for Infectious Diseases and Microbiology Laboratory Services, Westmead, NSW under accession numbers 80-19-303-6205 to 80-19-303-6209, 80-19-303-6211, 80-19-303-6213, 80-19-303-6215 to 80-19-303-6217, and 80-19-248-6803. Phylogenetic trees and alignment matrices have been deposited at TreeBase and are available under study accession URL http://purl.org/phylo/treebase/phylows/study/TB2:S26458. All other data are available in the manuscript or supplementary materials.
