# Publisher Correction: Cross-continental emergence of *Nannizziopsis barbatae* disease may threaten wild Australian lizards

**DOI:** 10.1038/s41598-021-86463-0

**Published:** 2021-03-19

**Authors:** Nicola R. Peterson, Karrie Rose, Stephanie Shaw, Tim H. Hyndman, Lynne Sigler, D. İpek Kurtböke, Josh Llinas, Bethan L. Littleford‑Colquhoun, Romane Cristescu, Celine Frère

**Affiliations:** 1 School of Science, Technology and Engineering, University of the Sunshine Coast, Sippy Downs, QLD 4556 Australia; 2 Australian Registry of Wildlife Health, Taronga Conservation Society Australia, Mosman, NSW 2088 Australia; 3 University of Queensland, Avian and Exotic Pet Service, Gatton, QLD 4343 Australia; 4 School of Veterinary Medicine, Murdoch University, Murdoch, WA 6150 Australia; 5 Faculty of Agricultural, Life, and Environmental Sciences, University of Alberta, Edmonton, AB T6G 2P5 Canada; 6 The Unusual Pet Vets, Jindalee, QLD 4074 Australia

Correction to: *Scientific Reports* https://doi.org/10.1038/s41598-020-77865-7, published online 01 December 2020

This Article contains an error in Reference 6, which is incorrectly given as:

Locxh, J. M. et al. Snake fungal disease: An emerging threat to wild snakes. Philos. Trans. R. Soc. B Biol. Sci. 20, 20. https://doi.org/10.1098/rstb.2015.0457 (2016).

The correct reference is listed below:

Lorch, J. M. et al. Snake fungal disease: An emerging threat to wild snakes. Philos. Trans. R. Soc. B Biol. Sci. 20, 20. https://doi.org/10.1098/rstb.2015.0457 (2016).

