# Supplementary material for: Cross-continental emergence of Nannizziopsis barbatae disease may threaten wild Australian lizards
Source: Sci Rep. 2020 Dec 1;10:20976. doi: 10.1038/s41598-020-77865-7 (PMC7708475; doi:10.1038/s41598-020-77865-7)
Supplement: Supplementary file 1 — Supplementary Information. [file 41598_2020_77865_MOESM1_ESM.docx]

**Scientific Reports**

Supplementary Materials:

**Cross-continental emergence of *Nannizziopsis barbatae* disease may threaten wild Australian lizards**

**Authors:** N. R. Peterson^1*^**^†^**, K. Rose^2^**^†^**, S. Shaw^3^, T. H. Hyndman^4^, L. Sigler^5^, D. İ. Kurtböke^1^, J. Llinas^6^, B. L. Littleford-Colquhoun^1^, R. Cristescu^1^, C. Frere^1^

**Affiliations:**

^1^University of the Sunshine Coast, Genecology Research Centre, School of Science and Engineering, Sippy Downs, Queensland, 4556, Australia.

^2^Australian Registry of Wildlife Health, Taronga Conservation Society Australia, Mosman, New South Wales, 2088, Australia

^3^University of Queensland, Avian and Exotic Pet Service, Gatton, Queensland, 4343, Australia

^4^Murdoch University, School of Veterinary Medicine, Murdoch, Western Australia, 6150, Australia

^5^University of Alberta, Faculty of Agricultural, Life, and Environmental Sciences, Edmonton, Alberta, T6G 2P5, Canada

^6^The Unusual Pet Vets, Jindalee, Queensland, 4074, Australia

*Materials & Correspondence to: nicola.peterson6@gmail.com

**^†^**These authors contributed equally to this work

**This file includes:**

Figs. S1 to S3

Tables S1 to S6


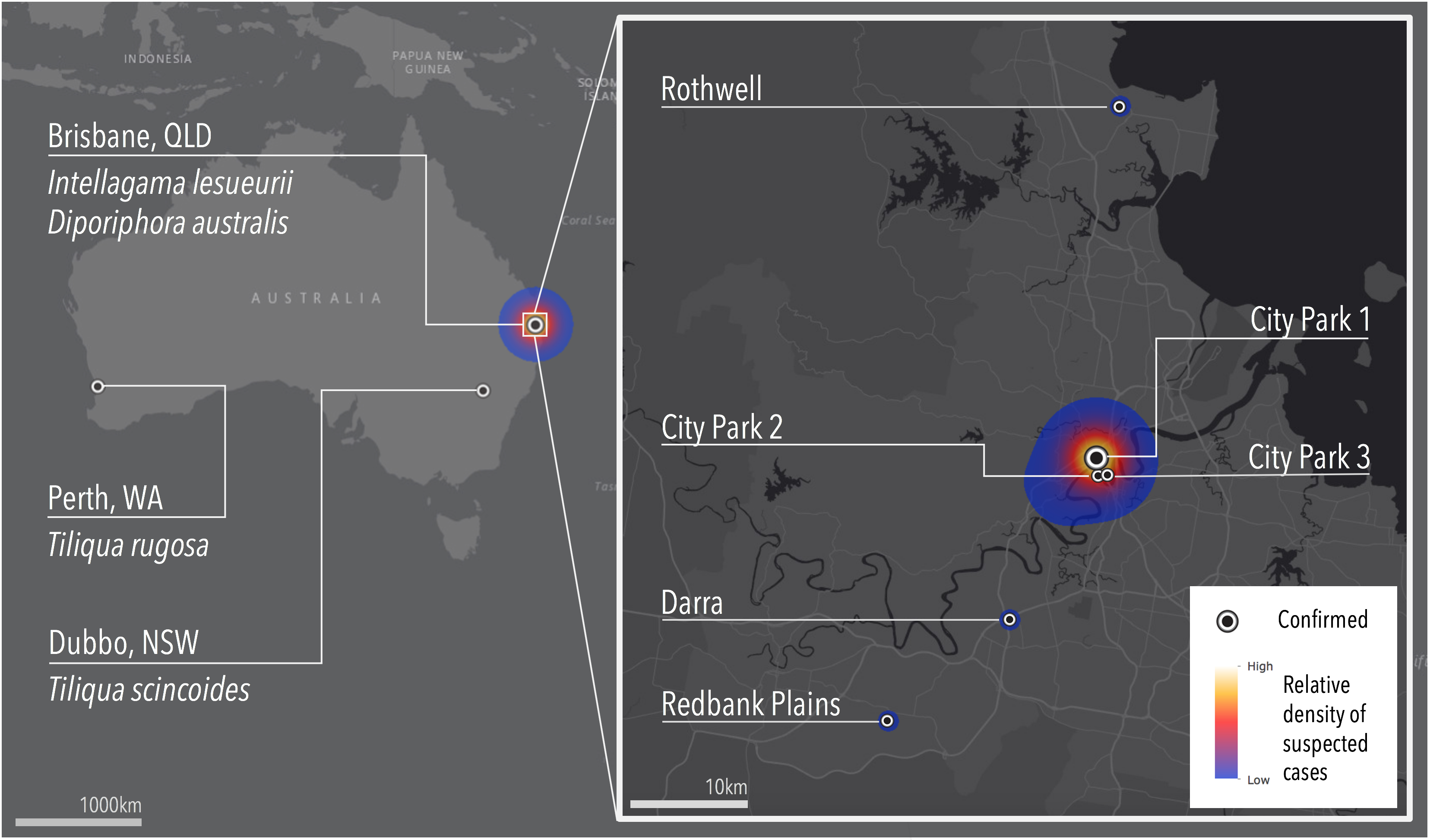


**FIG S1** Locations of free-living lizards with severe dermatitis. Using histology, culture, and molecular methods, *Nannizziopsis* *barbatae* was identified as the etiologic agent in all cases investigated. [Basemap generated using ArcGIS Online^57^]

| **Table S1** Summary of diagnostic tests used to detect *Nannizziopsis* species from cutaneous lesions of free-ranging and captive Australian lizards with severe dermatomycosis | | | | | | | |
| --- | --- | --- | --- | --- | --- | --- | --- |
| Case ID | Host species | Location | Date | Histology | Culture | *Nannizziopsis*-specific PCR (by sample type) | |
| EWD001 | Eastern water dragon (*Intellagama lesueurii*) | Redbank Plains, QLD  (-27.650800, 152.853700) | May, 2013 | + | + * | *Fungal isolate* | + |
| EWD002 | Eastern water dragon (*Intellagama lesueurii*) | Brisbane, QLD  City Park 1  (-27.463000, 153.019700) | Dec, 2017 | + | + ^‡^ | *Skin swab* | + |
|  |  |  |  |  |  | *Cutaneous lesion* | + |
|  |  |  |  |  |  | *Fungal isolate* | + |
| EWD003 | Eastern water dragon (*Intellagama lesueurii*) | Brisbane, QLD  City Park 1  (-27.463000, 153.019700) | Nov, 2018 | + | + ^‡^ | *Skin swab* | + |
|  |  |  |  |  |  | *Cutaneous lesion* | + |
|  |  |  |  |  |  | *Fungal isolate* | + |
| EWD004 | Eastern water dragon (*Intellagama lesueurii*) | South Brisbane, QLD  City Park 2  (-27.476000, 153.021100) | Nov, 2018 | + | + ^‡^ | *Skin swab* | + |
|  |  |  |  |  |  | *Cutaneous lesion* | + |
|  |  |  |  |  |  | *Fungal isolate* | + |
| EWD005 | Eastern water dragon (*Intellagama lesueurii*) | Brisbane, QLD  City Park 1  (-27.463000, 153.019700) | Nov, 2018 | + | + ^‡^ | *Skin swab* | + |
|  |  |  |  |  |  | *Cutaneous lesion* | − |
|  |  |  |  |  |  | *Fungal isolate* | + |
| EWD006 | Eastern water dragon (*Intellagama lesueurii*) | Brisbane, QLD  City Park 3  (-27.475100, 153.029600) | Nov, 2018 | + | + ^‡^ | *Skin swab* | + |
|  |  |  |  |  |  | *Cutaneous lesion* | + |
|  |  |  |  |  |  | *Fungal isolate* | + |
| EWD007 | Eastern water dragon (*Intellagama lesueurii*) | Brisbane, QLD  City Park 1  (-27.463000, 153.019700) | Nov, 2018 | + | + **^†^** | *Skin swab* | + |
|  |  |  |  |  |  | *Cutaneous lesion* | + |
|  |  |  |  |  |  | *Fungal isolate* | + |
| EWD008 | Eastern water dragon (*Intellagama lesueurii*) | Darra, QLD  (-27.578000, 152.950000) | March, 2019 | + | + **^†^** | *Skin swab* | + |
|  |  |  |  |  |  | *Cutaneous lesion* | + |
|  |  |  |  |  |  | *Fungal isolate* | + |
| EWD009 | Eastern water dragon (*Intellagama lesueurii*) | Brisbane, QLD  City Park 1  (-27.463000, 153.019700) | April, 2019 | + | + **^†^** | *Skin swab* | + |
|  |  |  |  |  |  | *Cutaneous lesion* | + |
|  |  |  |  |  |  | *Fungal isolate* | + |
| TRH001 | Tommy roundhead dragon  (*Diporiphora australis*) | Rothwell, QLD  (-27.210000, 153.035000) | July, 2019 | + | ND | *Cutaneous lesion* | + |
| EBT001^§^ | Eastern blue tongue skink  (*Tiliqua scincoides scincoides*) | Dubbo, NSW  (-32.280000, 148.580000) | August, 2019 | ND | + * | *Fungal isolate* | + |
| CBT001^C^ ^§^ | Centralian blue tongue skink  (*Tiliqua multifasciata*) | Lilydale, VIC | July, 2019 | + | + * | *Cutaneous lesion* | + |
| SBS001 | Shingleback skink  (*Tiliqua rugosa*) | Perth, WA (-31.960900, 115.832200) | March, 2020 | + | − * | *Cutaneous lesion* | + |
| + Positive for *Nannizziopsis* species  − Negative for *Nannizziopsis* species  ND, not determined  ^C^Captive animal, all others free-living  *Fungal isolation performed using cutaneous lesion segments plated on a selective medium^25^  **^†^**Fungal isolation performed using a serial dilution method developed for this study, as described in Materials and Methods  ^‡^Fungal isolation performed using serial dilution methodology following negative result using previously described methods  ^§^Evidence of *Nannizziopsis* also confirmed by lesion impression smear | | | | | | | |

| **Table S2** Similarities between sequences obtained from fungal isolates in this study and the nearest identity sequences publicly available from GenBank. | | | |
| --- | --- | --- | --- |
|  | Pairwise identity (%)  (matching nucleotides/sequence length in base pairs) | | |
|  | ITS | 28S | β-tubulin |
| Case ID | *N. barbatae*  (JF323871.1^T^ ) | *N. crocodili*  (MT478064.1) | *N. pluriseptata*  (HF547890.1^T^ ) |
| EWD001* | 98.6  (551/559) | 99.0  (522/527) | 88.5  (330/373) |
| EWD002* | 98.9  (277/280) | 99.0  (522/527) | 88.2  (329/373) |
| EWD003* | 98.9  (277/280) | 99.0  (522/527) | 87.7  (327/373) |
| EWD004* | 99.6  (279/280bp) | 98.9  (518/524) | 88.5  (330/373) |
| EWD005* | 98.9  (277/280) | 99.0  (522/527) | 88.2  (329/373) |
| EWD006* | 98.9  (277/280) | 99.0  (522/527) | 88.5  (330/373) |
| EWD007* | 98.9  (277/280) | 99.0  (522/527) | 87.7  (327/373) |
| EWD008* | 100  (240/240) | 99.0  (522/527) | 88.5  (330/373) |
| EWD009* | 98.9  (276/279) | 99.0  (522/527) | 88.2  (329/373) |
| TRH001**^†^** | 99.5  (219/220) | NS | NS |
| EBT001* | 100  (194/194) | 99.0  (522/527) | NS |
| CBT001**^†^** | 100  (220/220) | NS | NS |
| SBS001**^†^** | 100  (220/220) | NS | NS |
| ^T^ Sequence from ex-type strain  *Sequence obtained from fungal isolate DNA extract amplicons  **^†^** Sequence obtained from cutaneous lesion DNA extract amplicons  NS Not amplified or sequenced | | | |

| **Table S3** Presentation and gross pathology of Australian lizards with severe dermatomycosis associated with *Nannizziopsis* *barbatae* | | | | | | | |
| --- | --- | --- | --- | --- | --- | --- | --- |
| **Case ID** | **Signalment** | **Presentation** | **Treatment** | **Outcome** | **Distribution of skin lesions** | **% skin affected** | **Other Findings** |
| **EWD001** | Female, adult | Emaciated, lethargic | Oral itraconazole, disinfectant baths  (22 days) | Euthanized | Throat, ventrolateral abdomen, limbs, tail | 60 | Nil |
| **EWD002** | Female, adult | Emaciated, lethargic | Nil | Euthanized | Ventral abdomen, limbs, tail | 25 | Nil |
| **EWD003** | Male, adult | Thin | Nil | Euthanized | Ventral abdomen, limbs, tail | 25 | Necrosis and loss of tail tip and five digits |
| **EWD004** | Female, adult | Emaciated, dehydrated, lethargic | Nil | Euthanized | Throat, dorsal posterior body, abdomen, limbs, tail | 30 | Swollen anterior limb; partial hind limb amputation |
| **EWD005** | Female, adult | Emaciated, dehydrated, lethargic | Nil | Euthanized | Ventrolateral abdomen, limbs | 30 | Cutaneous ulceration; partial loss of one digit; swollen anterior limb |
| **EWD006** | Female, adult | Thin | Nil | Euthanized | Throat, ventral abdomen, limbs, tail | 25 | Necrosis and partial to complete loss of five digits; gravid with 10 well-developed eggs |
| **EWD007** | Male, adult | Emaciated, dehydrated, lethargic | Nil | Euthanized | Dorsolateral and ventral abdomen, limbs | 10 | Necrosis and loss of two digits; injuries to anterior limb and jaw |
| **EWD008** | Male, subadult | Emaciated, dehydrated | Nil | Died | Throat, ventral and dorsolateral abdomen, limbs | 40 | Necrosis and loss of two digits |
| **EWD009** | Male, adult | Thin, dehydrated | Nil | Euthanized | Upper mandible, rostrum, nostril, limbs, tail | 10 | Hyperkeratosis causing complete unilateral nasal obstruction |
| **TRH001** | Male, subadult | Thin, lethargic | Nil | Euthanized | Upper and lower mandibles | 10 | Nil |
| **EBT001** | Sex un-determined, adult | Good body condition, alert | Debridement, surgical removal of limb, topical terbinafine, iodine baths (14 days) | Lesions resolved, released to wild prior to confirmation of *Nannizziopsis* sp. infection. Lost to follow up. | Limbs, ventral right shoulder | 10 | Necrosis and loss of digits and tail tip; partial hind limb amputation |
| **CBT001^C^** | Male, adult | Thin, lethargic | Debridement, chlorhexidine bath, oral itraconazole (10 days) | Died | Limbs, ventral abdomen and pelvis | 30 | Nil |
| **SBS001** | Male, adult | Thin, lethargic | Nil | Euthanized | Throat, ventral abdomen, pelvis, tail | 40 | Nil |
| **^C^** Captive animal, one of five affected *Tiliqua multifasciata* housed together, three of which died and two that were euthanized | | | | | | | |

##### **
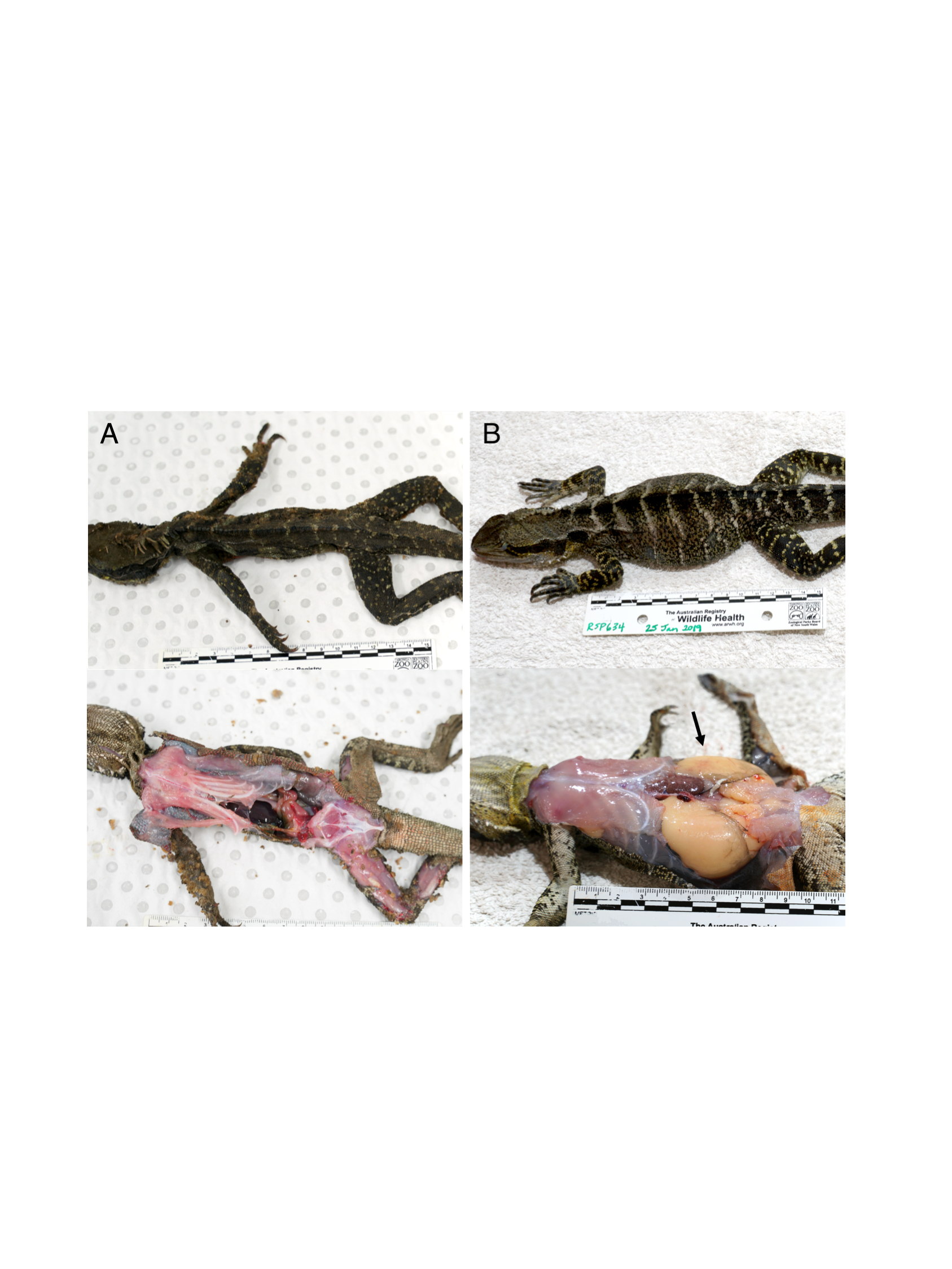
**

###### **FIG S2** Comparative body condition of Eastern water dragons affected and unaffected by *Nannizziopsis* *barbatae*-associated dermatomycosis. (A) Case EWD008 showing reduced muscle mass and absence of visceral fat stores; confirmed positive for *N. barbatae* by histopathology, culture, and PCR/sequencing of fungal isolate (B) Adult female, killed by vehicle strike and collected opportunistically; skin swab and cutaneous tissue were PCR-negative for *Nannizziopsis* species. Arrow indicates coelomic fat bodies.

| **Table S4** Histopathology of Australian lizards with severe dermatomycosis associated with *Nannizziopsis barbatae* | | | | | | |
| --- | --- | --- | --- | --- | --- | --- |
| **Case ID** | **Hyperkeratosis** | **Epidermal hyperplasia** | **Necrosis** | **Dermal inflammation** | **Depth of**  **fungal hyphae** | **Other findings** |
| **EWD001** | 3 | 3^e^ | 2 - epidermal | 2 L, H, P | Stratum spinosum | Epidermal intracytoplasmic inclusion bodies^*^; pancreatic granuloma (parasitic); granulomatous oophoritis; myodegeneration; cataract |
| **EWD002** | 3 | 2 | 2 - epidermal | 1 H, L | Stratum spinosum | Urate nephrosis; visceral gout |
| **EWD003** | 3 | 3^e^ H | 2 - epidermal | 1^e^ H | Stratum spinosum | Epidermal intracytoplasmic inclusion bodies^*^; thyroid hyperplasia |
| **EWD004** | 3 | 3^e^ H | 2 - epidermal | 1 H, L | Stratum spinosum | Nil |
| **EWD005** | 3 | 3^e^ H | 2 - epidermal | 1 H, L | Stratum spinosum | Membranous glomerular nephropathy; granulomatous glossitis; granulomatous oophoritis; hepatic necrosis |
| **EWD006** | 3 | 3^e^  H | 2 - epidermal | 1^e^ | Stratum spinosum | Epidermal intracytoplasmic inclusion bodies^*^ |
| **EWD007** | 3 | 3^e^  H | 2 - epidermal | 1^e^ H, L, M, P | Stratum spinosum | Epidermal intracytoplasmic inclusion bodies^*^; dermal granuloma (bacterial); urate nephrosis; membranous glomerular nephropathy; hepatic atrophy; gastric granuloma (helminth); gastric erosion; osteolysis; myodegeneration |
| **EWD008** | 3 | 3^e^ H | 3 - epidermal,  ulcerative  2 - dermal | 2^g^ H, L, M, P | Dermis | Epidermal intracytoplasmic inclusion bodies^*^; oral and tracheal petechial hemorrhage and necrosis; esophagitis with hemorrhage and necrosis; renal tubular epithelial necrosis; myodegeneration |
| **EWD009** | 3 | 3^e^ H | 2 - epidermal | 1^e, g^ H, L, M, P | Stratum spinosum | Epidermal intracytoplasmic epidermal inclusion bodies^*^; renal tubular necrosis and urate nephrosis; epidermal inclusion cyst; gastric mucosal necrosis and erosion with intracytoplasmic inclusions |
| **TRH001** | 2 | 3 H | 3 - epidermal,  ulcerative  1 - dermal | 2 H, L, M | Stratum spinosum | Nil |
| **CBT001** | 2 | 1 H | 3 - epidermal,  ulcerative  3 - dermal | 2 H | Stratum corneum | Nil; internal organs not available for histology |
| **SBS001** | 2 | 1 H | 3 - epidermal,  ulcerative  3 - dermal  3 - bone (osteoderm) | 2^e^ H, L | Stratum corneum | Cystic urinary calculi and chronic interstitial fibrosis; splenopancreatic vasculopathy and vasculitis |
| Findings graded on a scale of 0-3; 0-Normal, 1-Mild, 2-Moderate, 3-Severe  Cellular infiltrates: H-heterophilic, M-macrophages, L-lymphocytic, P-plasmocytic  ^e^-intercellular edema  ^g^-granulomatous  ^*^ PCR-negative for poxvirus and papillomavirus | | | | | | |

*
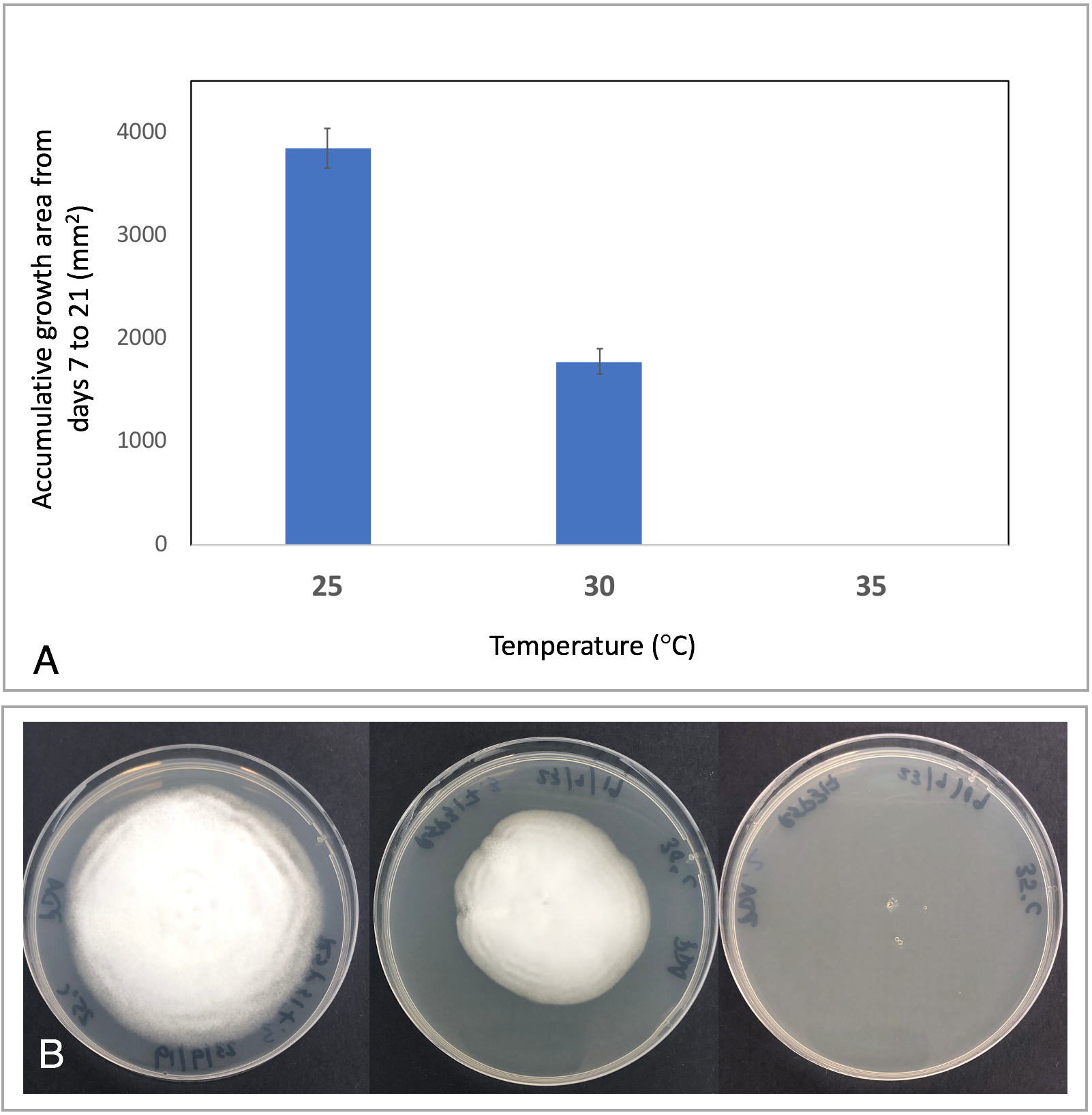
*

###### **FIG S3** Effect of temperature on growth of *Nannizziopsis barbatae*, isolated from Eastern water dragon cutaneous lesions. (A) Bars represent mean area of colony growth between days 7 to 21, at 25 °C, 30 °C, and 35 °C; error bars indicate standard error of the mean. (B) Representative colony (EWD003) from each temperature at day 21, from left to right: 25 °C, 30 °C, 35 °C.

| **Table S5** Additional microorganisms cultured from cutaneous lesions of lizards from which *Nannizziopsis barbatae* was confirmed | | |
| --- | --- | --- |
| **Case ID** | **Bacteria** | **Fungi** |
| EWD001 | *Staphylococcus epidermidis* | None |
| EWD002 | Mixed growth, including *Enterobacter cloacae* | *Aspergillus* and *Candida* spp. |
| EWD003 | - | *Aspergillus*, *Candida*, and *Fusarium* spp. |
| EBT001 | - | Mixed growth, unspecified |
| CBT001 | Mixed growth, including *Streptococcus* sp., *Corynebacterium*, *Staphylococcus* sp. and moderate growth of *Stenotrophomonas maltophilia* | Mixed growth, unspecified |
| SBS001 | - | *Fusarium* spp. |

###### -, not determined

| **Table S6** Publicly available *Nannizziopsis* sequences included in phylogenetic analyses | | | | | | |
| --- | --- | --- | --- | --- | --- | --- |
| Species | Isolate ID | Origin | GenBank Accession No. | | | Ref.  No. |
|  |  |  | ITS | 28S | β-tubulin |  |
| *Nannizziopsis*  *arthrosporioides* | **UTHSC R4263 ^T^**  UAMH 11232 | *Physignathus* sp., USA  Leopard gecko,  *Eublepharis macularius*, USA | HF547872.1  KF477202.1 | HF547857.1  - | HF547886.1  - | ^27^  ^8,13^ |
| *N. barbatae* | **UAMH 11185 ^T^** | Coastal bearded dragon, *Pogona barbata*, Australia | JF323871.1 | - | - | ^8,20^ |
| *N. chlamydospora* | **UTHSC 04-2056 ^T^**  UTHSC 06-1419 | Bearded dragon*, P. vitticeps*, USA  *P. vitticeps*, USA | HF547870.1  HF547871.1 | HF547854.1  HF547855.1 | HF547880.1  HF547882.1 | ^27^  ^27^ |
| *N. crocodili* | UAMH 9665  UAMH 9908  CIDMLS 80-15-229-5402 | Saltwater crocodile, *Crocodylus porosus*, Australia, Nth Qld  Saltwater crocodile, *Crocodylus porosus*, Australia, Nth Qld  Freshwater crocodile, *Crocodylus johnstoni*, Australia, SE Qld | KF477203.1  KF477205.1  MT478063.1 | -  -  MT478064.1 | -  -  - | ^8,10^  ^8,10^  ^9^ |
| *N. dermatitidis* | **UAMH 7583 ^T^** | Jewel chameleon (*Furcifer lateralis*), Canada | KF477200.1 | - | - | ^8^ |
| *N. draconii* | **CCFVB CH12 ^T^** | *Pogona vitticeps*, Spain | EU883993.1 | HF547856.1 | HF547884.1 | ^27^ |
| *N. guarroi* | UTHSC 06-3993  **CBS 124553 ^T^**  UAMH 10211  UAMH 10409 | *Agama agama*, USA  *Iguana iguana*, Spain  *P. vitticeps*, USA, WI  *P. vitticeps*, USA, NY | HF547875.1  EU018451.1  KF477207.1  KF477209.1 | HF547866.1  MH874904.1  -  - | HF547898.1  HF547896.1  -  - | ^27^  ^14^  ^8,19^  ^8^ |
| *N. pluriseptata* | UTHSC 10-1045 | Skink, *Eumeces inexpectatus,* USA | HF547874.1 | HF547859.1 | HF547890.1 | ^27^ |
| *N. vriesii* | UAMH 3526  **UAMH 3527 ^T^**  (CBS 407.71) | Soil, USA  Teiid lizard, *Ameiva* sp., Netherlands | KF477197.1  KF477198.1 | -  KC989736.1 | -  HF547894.1 | ^8^  ^8,58^ |
| *N. hominis* | **UAMH 7859 ^T^** (UTHSC 94-1427)  UAMH 9852 (UTHSC 00-1109) | Human, HIV^+^, USA  Human, USA | KF477215.1  KF477216.1 | -  - | -  - | ^8^  ^8^ |
| *N. infrequens* | **UAMH 10417 ^T^** | Human, HIV^+^, USA | AY744467.1 | - | - | ^8,59^ |
| **^T^** Type Strain | | | | | | |
